# Music and Early Language Acquisition

**DOI:** 10.3389/fpsyg.2012.00327

**Published:** 2012-09-11

**Authors:** Anthony Brandt, Molly Gebrian, L. Robert Slevc

**Affiliations:** ^1^Shepherd School of Music, Rice UniversityHouston, TX, USA; ^2^Psychology, Language and Music Cognition Lab, University of MarylandCollege Park, MD, USA

**Keywords:** music, language, language acquisition, childhood development, musical development, music cognition, definition of music, emergent modularity

## Abstract

Language is typically viewed as fundamental to human intelligence. Music, while recognized as a human universal, is often treated as an ancillary ability – one dependent on or derivative of language. In contrast, we argue that it is more productive from a developmental perspective to describe spoken language as a special type of music. A review of existing studies presents a compelling case that musical hearing and ability is essential to language acquisition. In addition, we challenge the prevailing view that music cognition matures more slowly than language and is more difficult; instead, we argue that music learning matches the speed and effort of language acquisition. We conclude that music merits a central place in our understanding of human development.

## Introduction

Just as infants yearn to walk, they have an accelerated drive for language: by age three or four, a child has essentially become competent in his or her native language. While linguistic abilities will continue to be refined, all of the requisite skills for the processing and performing of speech have been acquired (Kuhl, 2004).

Music is recognized as a universal feature of human cognition: every healthy human is born with the ability to appreciate it. However, music’s role in human development is often viewed as ancillary and slower to mature. Wilson argues that “whereas language acquisition in children is fast and largely autonomous, music is acquired more slowly and depends on substantial teaching and practice.” As a result, he surmises that music appears “to be derived from language” (Wilson, 2012, p. 283). At its most extreme, Pinker (1997) has described music as “auditory cheesecake, an exquisite confection” without any biological utility.

In this paper, we present a contrasting view: spoken language is introduced to the child as a vocal performance, and children attend to its musical features first. Without the ability to hear musically, it would be impossible to learn to speak. In addition, we question the view that music is acquired more slowly than language (Wilson, 2012) and demonstrate that language and music are deeply entangled in early life and develop along parallel tracks. Rather than describing music as a “universal language,” we find it more productive from a developmental perspective to describe language as a special type of music in which referential discourse is bootstrapped onto a musical framework.

Several factors have clouded an understanding of the entanglement of music and language, especially in the very young. First, overly restrictive definitions of music often impose adult assumptions onto newborns. Second, music and language are often treated as largely independent systems whose convergence is dependent on factors such as musical training. Third, while language skill is typically measured against the adult population at large, musical skill is often measured against the expertise of professional musicians, leading to mismatched expectations that make music learning seem more arduous and time-consuming. In this paper, we address these issues and show evidence for a deep early entanglement of music and language and for development along largely similar lines.

## Defining Music

By adulthood, we all have well-developed ideas about music informed by our culture and individual taste. However, though we all feel we know what music is, it has proven remarkably hard to define. Cross and Morley (2008) cite two dictionary definitions of music: “the art of combining sounds of voices or instruments so as to achieve beauty of form and expression of emotion” and “the art or science of arranging sounds in notes and rhythms to give a desired pattern or effect.” They go on to state: “For contemporary musicologists and ethnomusicologists, these definitions are seriously unsatisfactory.” After reviewing other definitions, they conclude: “All these notions of music reveal themselves to be ideological constructs rooted in the workings of broader socio-economic and political forces, which change.” (Cross and Morley, 2008, pp. 6–7).

Operating without a clear, generalized definition of music has made scientific conclusions difficult to evaluate, as results cannot be standardized and conflicting data is harder to resolve. Creating such a definition is therefore our starting point for investigating the connection between music and early language acquisition.

A comprehensive scientific definition of music must take into account the following:

### Music varies across cultures

The world’s indigenous musical traditions are remarkably diverse and often contradict each other in both overt and subtle ways. The discrimination of consonance and dissonance has been cited as a human universal, with dissonance treated as displeasing (Fritz et al., 2009). However, Markoff (1975, p. 135) points out: “The parallel seconds, so widespread in Bulgarian polyphonic folk-singing, may on first hearing impress the listener as being extremely dissonant. Bulgarian folksingers, however, consider such interval combinations as representing a beauty which is likened to the ‘sound of ringing bells.”’ Playing in tune is something Westerners frequently take for granted: the beating created by out of tune notes is considered unpleasant. However, Javanese gamelan ensembles are deliberately de-tuned by small intervals to create beating; notes in perfect accord would be considered “wan and lifeless” (Tenzer, 1991, p. 33). Western musicians often emphasize purity of tone; noise characteristics are considered clumsy. In contrast, Japanese shakuhachi players highlight the noise qualities of their instrument: the sounds of breath and attack transients are considered deeply expressive (Tokita and Hughes, 2008).

Entrainment to a steady pulse is frequently cited as a universal feature of indigenous ensemble music (Cross, 2012). However, Mongolian khoomi throat singers chant in groups without a steady beat (Tuva, 1990). Japanese gagaku contains unpulsed sections and unmeasured pauses (Kyoto Imperial Court Music Orchestra, 1993; Tokita and Hughes, 2008). In heterophonic music, each voice or instrument embellishes a shared melody in its own way, creating a freely pulsed web. The heterophonic performance of psalms in the Scottish Hebrides is said to represent each singer’s “relation to God on a personal basis” (Knudsen, 1968, p. 10). There are many other examples of heterophonic music in traditions as diverse as Ukrainian, Arabic, and South American folk music (Lieneff, 1958; Aretz, 1967; Racy, 2004).

Cultural diversity is true even of basic musical attributes such as how frequencies are classified. In Western music, frequency is mapped onto space: pitches are “high” and “low” and go “up” and “down.” However, in Bali, pitches are “small” and “large”; to the Sayá people of the Amazon “young” and “old”; and to the Shona people of Zimbabwe “crocodile” (for low frequencies) and “those who follow crocodiles” (for high ones; Zbikowski, 2008; Eitan and Timmers, 2010).

Harwood (1976, p. 528) concludes: “Contemporary ethnomusicological research yields an unequivocal response to the question of whether musical structure is similar across cultures. The answer is… that similarities are rare and unsystematic.”

### Musical practice varies over time, even within the same tradition

If this article had been written 700 years ago, creating music with harmonic progressions would have been considered unorthodox: the practice was confined to a few locations in Western Europe (Cohen, 2001). Today harmony is so commonplace that ancient melodies originally performed alone or with a drone are now often “retrofitted” with chord progressions. As a result, ubiquity or popularity in a particular time or place is not a reliable metric. Because the arts are open-ended, a definition also has to allow for as yet unimagined possibilities.

### Music is often very ambiguous, even on an emotional level

Music is often described as a “language of emotions” (McGilchrist, 2009; Panksepp, 2009; Juslin and Sloboda, 2010). To many, music’s expressivity – unconstrained by literal meaning – is what makes it a “universal language” (Bernstein, 1976; Cross, 2005).

In order for this to be true, emotional readings should translate broadly across cultures. In Western music, one of the strongest examples of well established emotional attribution is the contrast between the major and minor modes: the major mode is associated with positive affects such as joy, triumph, and tranquility; the minor mode is associated with negative affects such as grief and anger. However, these emotional associations are culturally determined (see, e.g., Dalla Bella et al., 2001): for instance, the Jewish folk song “Hava Nagila” is in the minor mode, but the lyrics are “Let’s rejoice! Let’s rejoice and be happy!” (Rossi, 1998). Indeed, the major/minor expressive contrast was not even well established in Western Europe until late in the eighteenth century: Orfeo’s plaintive aria “Che faro senza Euridice” (“How will I fare without Euridice”) from Gluck’s opera *Orfeo and Euridice* (1752) is written in the “happy” key of C-Major (Gluck, 1992/1752). Although it remains possible that some emotionally relevant aspects of music (perhaps based on more general acoustic cues to emotion) are, in fact, cultural universals (Juslin and Laukka, 2003; Juslin and Västfjäll, 2008; cf. Bryant and Barrett, 2007), we cannot assume that this is the case.

Listening to music is very subjective; as a result, emotional responses are inconsistent and subject to revision. In 1868, a New England critic wrote about an orchestral performance: “It opened with eight bars of a commonplace theme, very much like Yankee Doodle…I regret to say that [what followed] appeared to be made up of the strange, the ludicrous, the abrupt, the ferocious, and the screechy, with the slightest possible admixture, here and there, of an intelligible melody” (Slonimsky, 1965, p. 52). The work? The choral Finale of Beethoven’s *Ninth Symphony*, now celebrated as one of the Western canon’s most emotionally gripping works.

Ascertaining a creator’s intent is speculative. Although Leonard Bernstein felt confident his feelings matched those of Beethoven, he acknowledged “We’ll never know and we can’t phone him up.” (Bernstein, 1976, p. 138). The Soviet composer Dmitri Shostakovich was able to fool the authorities with musical tributes deemed to be sincere that Shostakovich privately declared to be bitterly ironic. Commentators still debate whether certain of his works are patriotic or subversive (Fay, 1980). Music’s ambiguity can actually be an advantage in group interactions, enabling it “to be efficacious for individuals and for groups in contexts where language would be unproductive or impotent, precisely because of the need for language to be interpreted unambiguously” (Cross and Morley, 2008, p. 10). While we often *invest* music with emotion and connect deeply to it for that reason, there is too much inconsistency and uncertainty in both personal and cultural views to describe music as a “language of emotions.”

### Any sound can be treated musically

We often think of music as being performed by voices and melodic instruments. However, the palette of instrumental sounds extends all the way from the sine wave purity of a Western flute to the white noise of a maraca or cymbal crash. While melody is certainly a central feature of music in cultures throughout the world, it is not a prominent feature of many African and Asian drumming traditions, jazz drum solos, or in the extensive body of Western unpitched percussion works. Aboriginal didgeridoos produce different pitches when played by different players; performances rely on rhythm and timbre rather than melody to create musical interest (Tarnopolsky et al., 2005).

Nor is music limited to conventional instruments: the Vanuatu people of the Banks Islands perform “water drumming” by beating rhythmic patterns on waves (von Hornbostel, 1933). Percussionists’ instrumental battery typically includes brake drums, clay pots, and chimes made of shells, glass, and metal. George Antheil incorporated airplane propellers in his *Ballet mécanique*. The advent of recorded media gave rise to *musique concrete*, in which industrial and natural sounds were used as musical material by such composers as Pierre Schaeffer and Pierre Henry.

In order to satisfy all of the above requirements, we propose the following definition: music is creative play with sound; it arises when sound meets human imagination. The term “music” also implies a value placed on the acoustic parameters of envelope, frequency, and spectrum^1^ irrespective of any referential function. Musical content is created by the behavior and patterns of these parameters; it can apply to any activity involved with the production and human perception of sound. Any experience – from the strumming of a harp to the blowing of the wind – that involves the cognition of these basic attributes of sound is potentially *musical*. All of the historical features of music – whether they are steady pulse, recognizable melodies, familiar instruments, or even its treatment as an art-form – are higher order phenomena that are flexible, mutable, and culturally mediated.

Our definition puts no limitations on how sound is organized. There is no acoustic imperative that one sound *has* to lead to another. Having played a particular chord on the piano, there is nothing acoustically inevitable about what follows: I can play another chord, turn on a blender, begin chanting. As a result, how sounds are assembled and linked is extremely flexible. Music often promotes the illusion of flow through such higher order features as steady pulse, repeating patterns, imitation, and stepwise motion. However, flow can be manipulated and broken. In the Finale of his *Ninth Symphony*, Beethoven incorporates flashbacks to earlier movements. In his *Symphonies of Winds*, Igor Stravinsky alternates between contrasting passages as if cross-cutting in a film. The composer Karlheinz Stockhausen wrote a group of pieces in “moment form,” in which individual gestures are treated as independent and can be rearranged by the performer (Kramer, 1978).

Finally, we do not require that music exist within a temporal frame, with a clear beginning and end. In many indigenous cultures, musical behavior is woven into everyday life and not treated as a concert experience (Cross, 2012). In 1966, composer LaMonte Young and his partner Marian Zazeela created a “Dream House” in lower Manhattan. The house contained two oscillators that gradually went in an out of tune. For 4 years, Young and Zazeela lived in the house, inviting guests to make repeat visits. Jem Finer’s “Longplayer” is even more extreme: it is a 1000 year composition for Tibetan singing bowls. Thus far, Young’s dream house and Finer’s “Longplayer” are idiosyncratic. But with the widespread use of electronic media, it is not hard to imagine a future in which sound installations may exist all over the world.

Defining music as “creative play with sound” is both rigorous and inclusive, embracing the full range of musical expression across time and cultures. Icelandic folk song, whose vocal lines follow contours but not precise pitches, Balinese gamelan music, with its often speeding and slowing of pulse, and the open form pieces of Earle Brown, in which no two performances are alike, would all be recognized as music. Any more restrictive a definition risks being contradicted. McAllester writes: “Any student of man must know that somewhere, someone is doing something that he calls music but nobody else would give it that name. That one exception would be enough to eliminate the possibility of a real universal.” (McAllester, 1971, 379).

It is a central human impulse to develop every one of our biological capacities – often beyond its original function. We move – so we run, jump, and dance. We grasp – so we paint, hammer, and slice. We breathe – into flutes, molten glass, and balloons. Music is the natural outcome of a species that takes every facet of its behavior and explores, amplifies, and extends it: it is an on-going conversation between our biological infrastructure and the plasticity of our imaginations. An elemental definition of music that applies broadly across geography, cultures, and eras is vital because it highlights the dynamism of this creative process. Our abilities to engage in and appreciate “creative play with sound” and to consider sounds irrespective of referential function lie at the heart of early language acquisition.

## The Music of Speech

Language is commonly defined as a symbolic medium for communication, with a lexicon of meanings and syntax for organizing its propositions^2^. We don’t just speak to be heard, we speak to be *understood* – to make declarations of love, order a meal, and ask for directions. But while speech is symbolic, sound is the bearer of its message.

Depending on how one listens, the same stimuli can be perceived as language or music. When one repeatedly listens to the same looped recording of speech, it can begin to sound like singing (Deutsch et al., 2011; Tierney et al., in press): as attention to meaning is satiated, the melodic features of prosodic inflection come to the fore. Conversely, sine wave speech, which tracks the formant frequencies of a spoken utterance without other acoustic attributes of natural speech, sounds like whistles to naïve listeners. However, when subjects are primed to listen for speech, the clips are clearly intelligible (Remez et al., 1981).

Within many cultures, there are gray areas between music and speech. The Ewe tribe in West Africa use talking drums to communicate between villages (Gleick, 2011) while “speakers” of Silbo Gomero use whistles to converse (Carreiras et al., 2005). In Cambodia, secular singing is typically accompanied by a fixed metrical pulse. Buddhist practice argues against music for spiritual practice, so the religious chants, which are highly melodic, are nevertheless treated as speech, to be performed without a rhythmic accompaniment (Sam, 1998). Poetry, with its attention to such sonic features such as rhyming, assonance, alliteration, and metric design, is widely regarded as hovering between music and speech. Indeed, epic poems are often sung: the Finnish Kalevala is frequently performed as a “singing match” between two voices (Siikala, 2000).

As adults, we process “canonical” speech and music differently: for example, speech and music show opposite patterns of hemispheric dominance, with speech processing relying more on the left hemisphere and music relying more on the right (e.g., Callan et al., 2006; Schön et al., 2010). Nevertheless, the neural regions underlying speech and music perception show significant overlap even in adults, with both types of stimuli recruiting a bilateral frontal-temporal network (Griffiths et al., 1999; Merrill et al., 2012). Furthermore, some differences between regions responsive to speech and song in adults is to be expected: over development, our brains become far more specialized in many domains (e.g., Durston et al., 2006; Scott et al., 2007). Although there is little work comparing neural responses to speech and music in infants, there is evidence that newborns show largely overlapping activation to infant directed speech and to instrumental music (Kotilahti et al., 2010) suggesting that processing differences in adult brains may have emerged gradually over the course of development.

It has been suggested that speech and music may have intrinsic differences in low-level auditory characteristics that require different types of aural processing: for instance, some have proposed that speech includes very rapidly changing temporal features whereas music is made up primarily of pitch features varying over a longer time window (e.g., Zatorre et al., 2002). However, speech and music turn out to be closely related in this regard. Perception of temporal changes on the order of 25–50 ms is crucial for the extraction of segmental and phonemic information from the speech signal (Tallal and Piercy, 1973; Rosen, 1992; Telkemeyer et al., 2009). Perception within this small time window is also crucial for instrument recognition. No musical instrument begins with a stable frequency: there is always an onset of noise, caused by the initial impulse that sets the sound in motion. This burst of noise is crucial for timbre perception (Hall, 1991). As a result, the same temporal acuity is required to process both speech and musical timbre (Shepard, 1980; Hukin and Darwin, 1995; Robinson and Patterson, 1995). This is true whether many instruments are playing or just one: Stepanek and Otcenasek (2005) demonstrate remarkable variety in the sounds of a violin, based on register, articulation, and fingering. Thus, both the perception of musical timbres and phonemes rely on rapid temporal processing.

In addition, languages vary in the extent to which they rely on these rapid phonemic cures: some African dialects incorporate as many as 150 separate phonemes, while others, such as Hawaiian, use fewer than 20 (Maddieson, 1984). Similar to speakers of Silbo Gomera, the Pirahã people of the Amazon can converse without phonemes with a humming language; using “intonation, timing, syllable patterns, and stress” and whistling with “no apparent limits as to the quantity, complexity, or kind of information transmitted.” Although whistling and humming languages are rare, they are an important reminder that language performance is not confined to timbral control (Everett, 1985, 413–414).

Even in languages with rich phonemic inventories that would presumably rely heavily on timbral processing, only a small set of speech sounds actually require resolution on very rapid timescales (McGettigan and Scott, 2012). Instead, the primitives of speech perception might be on a longer timescale corresponding roughly to syllables (e.g., Morillon et al., 2010). Of course, music also relies on analysis over longer time windows: in an instrument such as a flute or piano, the noisy onset resolves into a sustained pitch – the basis of musical melody. This pitched sustain, which takes longer for the ear to measure, is an important part of speech perception as well. This is most clear in tone languages (the most widely spoken being Mandarin Chinese), where pitch is lexically contrastive. In the African language of Kele, the phrase “*alamhaka boili*” has two very different meanings depending on its pitch inflection: it can either mean “He watched the river-bank” or “He boiled his mother-in-law” (Gleick, 2011, 23).

Even in non-tone languages, pitch is an important feature of speech performance. Accented syllables help to parse streams of speech into individual words (e.g., Cutler and Norris, 1988). Pitch inflection is also a primary feature of prosody, which conveys semantic structure and emotional affect. In English, declarative sentences generally end with a drop in pitch, whereas questions end with a rise. Prosody also influences meaning through variations in emphasis. There’s a famous joke in which an out-of-work actor is told he’s been hired as a sub for a Shakespeare performance. He has one line: “Hark! I hear the cannon roar.” He spends the afternoon rehearsing it: “Hark! *I* hear the cannon roar.” “Hark! I *hear* the cannon roar.” “Hark! I hear the *cannon* roar.” Variations in pitch and rhythm create his different line readings. Finally, he dresses in costume, is pushed on-stage and greeted with a loud explosion, to which he exclaims, “What the heck was that?”

Thus, both music and speech require aural resolution at similar time-scales. From a musical perspective, speech is a concert of phonemes and syllables, melodically inflected by prosody.

The congruence between speech and music at an atomic perceptual level became more evident with the advent of recorded media and electronic acoustic analysis. As a result, its effects have had a particularly notable impact on twentieth century music. Luciano Berio’s song cycle *Circles* employs a vast battery of percussion, which he uses to mimic various consonant sounds in the text. In one famous passage, the sibilants in the singer’s text are imitated by maracas and other shaken and rattling percussion instruments. The effect is of the text resonating among the percussion, which sustain and amplify the timbre of the words.

In Alvin Lucier’s *I Am Sitting in a Room*… for electronic tape, a recording of the composer reading a prepared text is broadcast through loudspeakers and rerecorded. As this process is looped, the natural resonance frequency of the room is enhanced and many of the recognizable features of the composer’s speech degrade. What is left at the end is the resonance frequency of the room pulsing with the stresses and cadence of Lucier’s speech. The lexical and syntactic features of language are stripped away, leaving behind the rhythmic residue of speech.

Other examples draw on the melodic aspects of prosody. In his album *Artist in Residence*, the jazz composer Jason Moran begins one track with an excerpt from a lecture by the artist Adrian Piper. Moran then repeats the clip, this time shadowing it with a piano solo matched to the rhythm and contour of Piper’s delivery. Finally, he replays the piano solo on its own and develops it into an extended improvization. Thus, Piper’s lecture provides the “melody” for the jazz solo (Moran, 2006).

The Berio, Lucier, and Moran works aren’t merely settings of text. The music is *drawn out* of the text: there is a direct translation or transformation of acoustic features of speech into a purely musical form.

Other composers and performers have made music out of phonemes treated for their musical value alone. Scat-singing is a type of jazz improvization in which wordless singing, nonsense syllables, and the occasional fragment of speech animate a vocal line, often to humorous effect. By marrying dexterous phonemic patterns with fluid and rhythmic musical lines, scat-singers highlight the human voice as a virtuosic and colorful musical instrument.

In his seminal work *Aventures*, Gyorgy Ligeti invents a glossary of nonsense syllables that serve the work’s intricate musical structure. Three singers perform their imaginary discourse with exaggerated prosody and a full battery of non-linguistic vocal sounds, including breathing, laughing, sighing, burping, and crying. What results is an intense portrait of human vocal communication devoid of referential meaning.

These creative examples make explicit the musicality of speech. Speech is sound. Its acoustic attributes – pitch, rhythm, and timbre – can serve strictly musical purposes.

Just as composers have made music out of speech, so too does every human voice. As adults, we learn to tone down the features of speech that do not contribute to meaning. In contrast, infants rely on a complete battery of musical information to learn speech: timbre, pitch, dynamic stress, and rhythm. There is no evidence that timbral information alone would be enough to acquire language; in fact, speech perception can be relatively successful even in the *absence* of timbral cues (Shannon et al., 1995). As the succeeding section will show, the comprehensive nature of the infant’s aural attention is a great asset in acquiring language: the infant’s attention to *all* of the musical features of speech provide a richer context for language induction.

## Music and Early Language Acquisition

In order to function in a community, basic speech has to be mastered by everyone. It needs to be understood even when delivered quickly and it needs to be capable of being performed even in moments of stress. All of these factors contribute to the design of this unique form of vocal performance.

But there is another critical feature of language: it needs to be learned by children. Many linguists and anthropologists emphasize that language as a symbolic system of expression is constrained by children’s ability to learn. Deacon writes: “The structure of a language is under intense selection pressure because in its reproduction from generation to generation, it must pass through a narrow bottleneck: children’s minds.” (Deacon, 1997, 110) Language is a compromise between what adults need to say and children’s ability to process and perform what they hear. And, crucially, what infants hear is, by the broad definition above, a form of music.

### Newborns’ sensitivity to musical sounds

Newborn infants’ extensive abilities in different aspects of speech perception have often been cited as evidence that language is innate (e.g., Vouloumanos and Werker, 2007). However, these abilities are dependent on their discrimination of the *sounds* of language, the most musical aspects of speech. We argue not that language has a privileged status in the newborn brain, but rather that *music* has a privileged status that enables us to acquire not only the musical conventions of our native culture, but also enables us to learn our native language. *Without the ability to hear musically, we would be unable to learn language*. Infants are famously able to discriminate the phonemes of all languages (Eimas et al., 1971; Werker and Tees, 1984; Dehaene-Lambertz and Dehaene, 1994), an ability that is evidence of sensitivity to timbre, as discussed above. Although newborns’ ability to discriminate different instrumental timbres has not yet been tested, infants are able to use timbre to segregate sound sequences into separate perceptual streams (McAdams and Bertoncini, 1997). If phonemic contrasts and instrumental timbral contrasts rely on the overlapping perceptual mechanisms in infants, one would expect similarly precocious abilities in instrumental timbre discrimination among newborns.

In addition to timbre, newborns are sensitive to the rhythmic components of language and can distinguish between languages based on their rhythmic characteristics (whether or not the contrast includes their native language; Nazzi et al., 1998). Newborns have a preference for their native language as well (Moon et al., 1993), however this has only been explored using languages from two different rhythmic classes. Because the ability to discriminate between two languages of the *same* rhythmic class (e.g., English and German) does not appear until 4 months of age (Nazzi et al., 1998; Gervain and Mehler, 2010), new borns may show a preference for *any* language belonging to the same rhythmic class as their native language. If so, then newborns may not prefer their native language *per se*, but rather the rhythmic characteristics of that language (cf. Friederici et al., 2007). Indeed, infants’ early attention to rhythm (e.g., Ramus and Mehler, 1999; Ramus et al., 1999) suggest that they are absorbing the sonic structure of their native language – its rhythms of stresses, its phonemic character – much in the same way that we listen to music.

Newborns can also discriminate a variety of other linguistic characteristics based on the musical aspects of language. For example, infants can distinguish the characteristic prosody (or melody) of their native language from others (Friederici, 2006). In fact, infants show electrophysiological evidence for discrimination of affective prosody even in the first few days of life (Cheng et al., 2012). Another piece of evidence that melodic abilities are important for language development comes from infant cries: the melodic complexity of crying increases over the first few months of life (Wermke and Mende, 2009), and infants who do not show such increasing melodic complexity also show poorer language performance 2 years later (Wermke et al., 2007). Infants can also discriminate individual words with different patterns of lexical stress (Sansavini et al., 1997), can detect acoustic cues that signal word boundaries (Christophe et al., 1994), can distinguish function from content words based on differing acoustic characteristics (Shi et al., 1999), and show sensitivity to prosodic boundaries in sentences (Pannekamp et al., 2006). Interestingly, word segmentation is, at first, based largely on rhythmic (stress) information, and only later do infants demonstrate sensitivity to other non-stress-based cues (Jusczyk et al., 1999). These findings suggest that these discrimination abilities may explain how infants solve the bootstrapping problem – i.e., how to connect the sounds to meaning. Put another way, infants use the musical aspects of language (rhythm, timbral contrast, melodic contour) as a scaffolding for the later development of semantic and syntactic aspects of language. Infants are not just listening for affective cues nor are they focused exclusively on meaning: they are listening for how their language is composed.

### Refinement of sound perception over development

Gradually, infants’ abilities become more refined and culture-specific. At 6 months of age, infants can still discriminate all the phonemic contrasts of the world’s languages (Cheour et al., 1998; Rivera-Gaxiola et al., 2005), although they show evidence of being attuned to the vowel sounds of their native language over other languages (Kuhl et al., 1992). Similarly, infants at this age do not show a perceptual bias for the music of their native culture: while Western adults more readily detect changes in melodies made up of pitches from the Western major/minor scale system than in melodies using Javanese scales, infants detect changes equally well in both scale systems (Lynch et al., 1990). This is also seen in the perception of musical meter: Western music overwhelmingly uses simple meters where the underlying beat pattern (regardless of the specific rhythm) is symmetrical and regular. A march goes along in groups of two, a waltz in groups of three and the two never mix (imagine trying to waltz to a piece of music constantly changing between 1-2-3, 1-2-3, and 1-2, 1-2 – you would trip over your partner’s feet). Non-Western cultures more commonly use this type of metrical mixing, or complex meters (e.g., Reinhard et al., 2001; Petrov et al., 2011). While Western adults have a harder time detecting changes in complex meters than in simple meters, 6 month old infants again can detect changes equally well (Hannon and Trehub, 2005a; note, though, that infants do develop a *preference* for the meter of their own culture earlier, even while they can accurately discriminate the meter of other cultures; Soley and Hannon, 2010).

**Figure 1:**
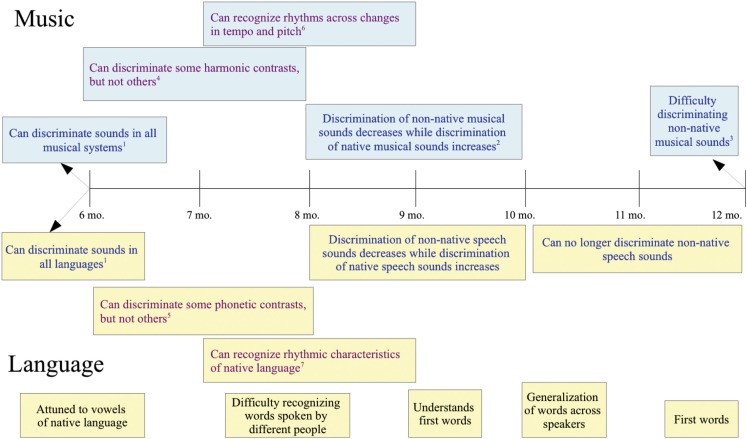
**Blue print denotes parallel development**. Purple print denotes related, but not analogous development. Black print denotes language-only development. See main text for citations not listed here. (1) Six-month olds can discriminate changes in Western and Javanese scales, can discriminate simple and complex meters, and can discriminate the phonemes of all languages. (2) Nine-month olds can detect pitch or timing changes more easily in strong metrical structures and more easily process duple meter (more common) than triple meter (less common; Bergeson and Trehub, 2006). (3) Twelve-month olds can better detect mistuned notes in Western scales than in Javanese scales and have more difficulty detecting changes in complex than simple meters. (4) Between 6 and 8 months, infants can discriminate consonant from dissonant intervals, but have difficulty discriminating between different consonant intervals (Schellenberg and Trainor, 1996). (5) Between 6 and 8 months, can no longer discriminate non-native vowel contrasts, but can still discriminate non-native consonant contrasts. (6) Trehub and Thorpe (1989). (7) At 7.5–8 months, English speaking infants show a bias for stress-initial words and are sensitive to prosodic and frequency cues to word order.

Between 6 and 12 months of age, infants’ linguistic and musical perception begins to become more specific to their native culture (Figure 1). This occurs earlier for vowel sounds than for consonants: 4–6 month old infants discriminate between non-native vowel contrasts, but 6–8 month old infants do not (Polka and Werker, 1994). In contrast, 6–8 month old infants still readily discriminate between non-native consonants and it is not until 10–12 months of age that most infants lose sensitivity to non-native contrasts (Werker and Tees, 1984)^3^. In addition to these changes in phonemic perception, by 9 months, infants have become especially sensitive to the stress pattern of their native language (see Jusczyk, 2000, for a review).

The same progression is seen in the perception of pitch and rhythm. By 12 months of age, Western infants show superior detection of mistuned notes in melodies from Western scales versus Javanese scales, just as adults do (Lynch and Eilers, 1992). This is in direct contrast to the 6-month old infants discussed earlier who showed no bias for their native scale structure. Likewise, 12-month old Western infants no longer easily process rhythm in complex meters in contrast to the 6-month old infants discussed above (Hannon and Trehub, 2005b). This perceptual narrowing, specific to an infant’s cultural experience, seems to be a domain-general phenomenon across perceptual modalities and is not specific to either music or language (Pascalis et al., 2002; Scott et al., 2007).

What kind of neurophysiological changes underlie this gradual specialization for the speech and musical sounds of one’s native culture? Presumably the brain becomes more specialized over development, reflecting a gradual emergence of adult-like networks (cf. Johnson, 2011). For example, “temporal voice areas” (Belin et al., 2000) in the superior temporal sulcus develop selectivity for voice identity and emotional prosody between 4 and 7 months of age (Grossmann et al., 2010; Blasi et al., 2011), corresponding nicely with the increasing sensitivity to culture-specific aspects of speech and music at that age.

As infants gradually become more sensitive to both the musical and linguistic sounds of their culture (and less sensitive to the characteristic sounds from other cultures), they also begin to lay the foundation for processing meaning and syntax. For instance, English speaking infants at 7.5 months show a preference for stress-initial words, which is the predominant stress pattern in English (Jusczyk et al., 1999). Eight-month old infants have become sensitive to the word order conventions of their native language, largely through their use of word frequency, and prosodic information (Weissenborn et al., 1996; Gervain et al., 2008; Nespor et al., 2008; Hochmann et al., 2010). Again, infants are first attuned to the musical aspects of language (stress patterns, prosody).

All of the aspects of language that an infant can perceive at birth and all of those aspects that are learned during the first year of life are *musical* by the definition of music that we are advocating. The aspects of language that differ the most from music come later:*the further removed a feature of language is from music, the later it is learned*. At around 9 months, infants show evidence of understanding their first words (Friederici, 2006). Once infants discover that words have referential meaning, semantic, and syntactic development takes over. Infants typically begin to talk between 11 and 13 months, experience a vocabulary growth spurt between 18 and 24 months, and reach the high point of their syntactic learning between 18 and 36 months (Friederici, 2006; Kuhl, 2010). From this point on, music and language likely proceed on relatively separate, but parallel, tracks as the musical aspects of language become secondary to its referential and discursive functions.

### Shared learning mechanisms

Infants learn the musical information of speech both by being spoken and sung to directly and by “overhearing” other language and music. Although all speech has musical aspects (see above), speech that is directed to infants is typically characterized by an even greater degree of musicality. This infant directed speech, or *motherese*, is relatively high pitched, slow, and rhythmic, with a larger pitch range and more exaggerated melodic contours than typical adult directed speech (Fernald, 1989). The use of motherese appears to be a cultural universal (Fernald, 1992; Falk, 2004) and intentions expressed in infant directed speech can be understood even across very different languages and cultures (Bryant and Barrett, 2007). Infants show a strong preference for infant directed speech (Fernald, 1985; Werker and McLeod, 1989), which seems to reflect the musical aspects of motherese as this preference remains (in 4 month old infants) even when the speech samples are filtered to remove lexical content while preserving the prosody (Fernald, 1985; see also Fernald and Kuhl, 1987).

Parents not only speak to their children in musical ways; singing also takes on a specific set of characteristics when directed to children (Trehub and Trainor, 1998). As with motherese, characteristics of infant directed song are shared across cultures and can be recognized cross-culturally (Trehub et al., 1993). Even infants of deaf parents (who presumably have heard relatively little infant directed speech or song) prefer this style of singing to adult directed music (Masataka, 1999).

Why do people speak and sing to infants in this especially musical way? It may be that infant directed speech and singing serves as an aid for language learning by capturing and engaging attention and communicating affective information (Fernald, 1989) and later by enhancing the important patterns in language (such as vowel categories and word divisions; e.g., Kuhl et al., 1997). Others have argued that the role of infant directed speech is not for learning *per se*, but rather serves as a vehicle for emotional communication (Trainor et al., 2000). Of course, infant directed speech might serve all of these purposes, and its role in language acquisition might change over the course of development: first playing an attentional and affective role, and later directing attention to linguistically relevant information (Fernald, 1992). Whatever its purpose, it is clear that a child’s first direct exposure to verbal communication frequently has heightened prosody, with the music, and meaning of speech all bound up together.

Human children are not just taught language directly: they learn it through immersion. Even at birth, infants have had considerable opportunities to learn: a fetus starts responding to sound at about the third trimester, and this time until about 6 months of age is a critical period of auditory perceptual development (Birnholz and Benacerraf, 1983; Graven and Browne, 2008). The sorts of sound that reach prenatal human ears differ from those conducted through air: high-frequency sounds are strongly attenuated, so prenatal stimulation is dominated by low-frequency energy. A fetus can thus detect vowels and musical pitches, but can perceive little of the auditory characteristics that will identify consonants or overtones (Gerhardt and Abrams, 2000).

Note, then, that the features of speech that a fetus is exposed to (and, thus, that an infant is most familiar with) are the more musical features of speech: low-frequency vowel sounds, pitch, and rhythm. Familiarity with these features may explain infants’ sensitivity to various aspects of sound at birth: newborns show a preference for their mothers’ voice (Mehler et al., 1978), for the sounds of their native language (Mehler et al., 1988; Moon et al., 1993), and can even recognize specific sound stimuli that they hear regularly *in utero*, be it a specific spoken passage (DeCasper and Spence, 1986) or a specific piece of instrumental music (James et al., 2002).

Learning from mere exposure continues after birth as well. Incidental learning of this kind is likely crucial for many aspects of development, including learning the sound structure of the language and music of one’s culture. When referring to language acquisition, this process is typically referred to as *statistical learning* (or sometimes *implicit learning*; cf. Perruchet and Pacton, 2006). Statistical learning refers to the (largely implicit) acquisition of structure in the environment, possibly reflecting relatively simple Hebbian learning processes in neural structures. This type of mechanism has seen considerable research in the realm of language development (see Romberg and Saffran, 2010, for a recent review) and has been considered as a mechanism of musical development as well (McMullen and Saffran, 2004; Hannon, 2010).

Statistical learning appears to be a very general phenomenon. It has been demonstrated not only for speech segmentation (Saffran et al., 1996; Mattys and Jusczyk, 2001) and phonetic category learning (Maye et al., 2002), but also for learning of patterns in tone sequences (Saffran et al., 1999), timbre sequences (Tillmann and McAdams, 2004), and even visual and tactile sequences (e.g., Conway and Christiansen, 2005). Interestingly, it seems that musical patterns (as defined here) are perhaps the most amenable to statistical learning: while adult participants can extract statistical regularities from visual and tactile sequences, they do so less well than with auditory sequences (Conway and Christiansen, 2005). Furthermore, a period of auditory deprivation in congenitally deaf children leads to impairments not only in auditory learning, but also in *visual* sequencing abilities (Conway et al., 2011), suggesting that musical learning may be critical not only to learn sound based statistics, but for the learning of sequential and temporal patterns in general (cf. the *auditory scaffolding hypothesis*; Conway et al., 2009). This account is not without its challenges; for example, recent evidence suggests that (adult) congenital amusics show impaired statistical learning of musical tone sequences despite normal statistical learning of speech sounds (Peretz et al., 2012). However it is likely that statistical learning of musical information – whether the learned patterns eventually are used in the service of speech or music – is a critical part of auditory development.

### Linked developmental deficits

Additional support for the idea that musical hearing is critical to language acquisition and ability comes from studies of children with language disorders and language delays. These children not only show difficulties with the musical aspects of language, but – very tellingly – they show impairments in music processing, too. Although the initial entanglement of music and language gradually unravels over the course of development, the fact that underlying deficits in musical hearing are associated with a variety of language impairments argues for the idea that although music and language grow apart, they are never truly separate in the brain.

Dyslexia, in particular, has been associated with more general auditory processing deficits. It is outside the scope of this paper to discuss these findings and theory at length (see Hämäläinen et al., in press, for a recent review), but it is worth noting some of the relevant research. One proposal is that dyslexia results from an underlying problem with rapid temporal processing (Tallal and Piercy, 1973), specifically of the quickly changing formant transitions that distinguish one consonant from another. Treatment programs that use exaggerated versions of these contrasts as well as musical stimuli (e.g., pitch glides) appear to improve reading ability by way of improvements in rapid temporal acuity (Merzenich et al., 1996; Tallal and Gaab, 2006; Gaab et al., 2007), although many studies of these treatments have not been well controlled (McArthur, 2009). Nevertheless, it is clear that dyslexia is associated with rapid temporal processing deficits, and given that phonemic distinctions are akin to the perception and discrimination of instrumental timbres, one would also expect dyslexics to have trouble distinguishing different instruments. Although little attention has been paid to timbre perception in dyslexics (or to timbre perception in general), there is some evidence that dyslexic children do show significantly impaired perception of timbre (Overy, 2000; Overy et al., 2003).

Dyslexic children may also be less sensitive to the amplitude modulations in speech (and other sounds) than normally developing children (Goswami et al., 2002). Indeed, dyslexic children’s perception of rise times and perceptual centers (the moment when a sound is perceived to occur) is impaired compared to typically developing children across a variety of language backgrounds (Goswami et al., 2002; Muneaux et al., 2004; Surányi et al., 2009). Interestingly, precocious readers show greater sensitivity to rise time than control subjects, and this sensitivity relates to reading progress (Goswami et al., 2002). Because sensitivity to rise time and perceptual centers is essentially sensitivity to the rhythm of language, dyslexic children are predicted to have difficulties with rhythmic tasks. In fact, dyslexic children do have trouble speaking in time with a metronome, tapping in time with a metronome, rhythm perception (saying whether two rhythms are the same or different), and tempo perception (Overy, 2000; Goswami et al., 2002; Huss et al., 2011).

Additional support for the idea that musical hearing is necessary for reading competency comes from longitudinal studies of newborns showing that cortical responses to speech and non-speech stimuli at birth are significant predictors of later dyslexia and reading problems (Molfese, 2000; Leppänen et al., 2010) and from a variety of other findings that pitch processing and other abnormal patterns of sound processing predict later reading ability (Leppänen et al., 2010, 2011). There is thus considerable evidence for auditory processing deficits in dyslexia, suggesting that developing competence in reading requires competence in musical hearing. Without an accurate perception of the musical elements of language, learning to read is very difficult, if not impossible.

Another language impairment that may reflect an underlying problem with musical hearing is Specific Language Impairment (SLI). SLI is a failure of normal language development despite normal intelligence and learning environment and an absence of hearing or emotional problems. As in dyslexia, infants that go on to develop SLI have difficulty with rapid temporal processing (Benasich and Tallal, 2002) and discrimination of rise time contrasts (Corriveau et al., 2007). Children later diagnosed with SLI also seem to have a reduced sensitivity to the duration of sounds, which can be seen as young as 2 months of age (Friedrich et al., 2004; Corriveau et al., 2007). The prototypical deficit in SLI is with syntactic processing, which extends to the processing of musical syntax as well (Jentschke et al., 2008). Finally, SLI is associated with impaired statistical learning of both speech stimuli and of non-linguistic tone sequences (Evans et al., 2009). These data suggest that many language learning deficits might be better understood as deficits in the processing complex auditory input (i.e., music). This broader definition may not only be more accurate, but may also help researchers and clinicians develop and advocate for more varied types of intervention.

### Parallels in music and language development beyond the first year

**Figure 2:**
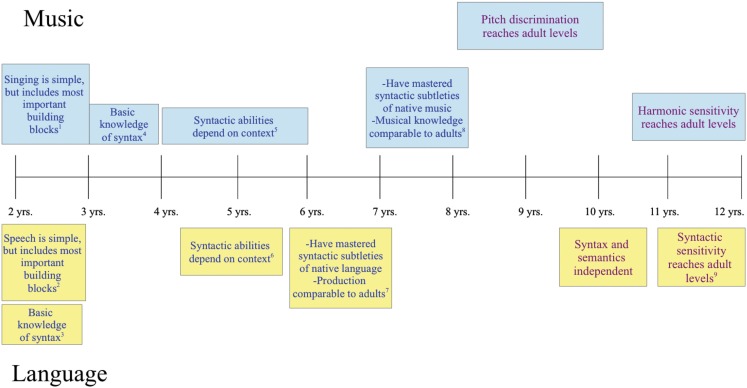
**Blue print denotes parallel development**. Purple print denotes related, but not analogous development. See main text for references. (1) Two-year olds can repeat brief, sung phrases with identifiable rhythm and contour. (2) Eighteen-month olds produce two word utterances; 2 year olds tend to eliminate function words, but not content words. (3) Two-year olds show basic knowledge of word order constraints. (4) Three-year olds have some knowledge of key membership and harmony and sing “outline songs.” (5) Four to six-year olds show knowledge of scale and key membership and detect changes more easily in diatonic melodies than in non-diatonic ones. Five-year olds show a typical electrophysiological response to unexpected chords (the early right anterior negativity, or ERAN), but do not detect a melodic change that implies a change in harmony. (6) At 5 years, processing of function words depends on semantic context and brain activation is not function-specific for semantic v. syntactic processing (unlike adults). (7) Six-year olds are able to speak in complete, well-formed sentences. (8) Seven-year olds have a knowledge of Western tonal structure comparable to adults’ and can detect melodic changes that imply a change in harmony. (9) Only after 10 years of age do children show adult-like electrophysiological responses to syntactic errors (Hahne et al., 2004).

The relationship between music and language continues past the first year of life (Figure 2). However, one challenge with comparing language and music development in later childhood is that, while speech ability is measured against the general population, musical ability is often implicitly measured against the virtuosity and expertise of professional musicians. This has contributed to the perception that, whereas language is an innate skill, music is a “gift” and much slower to mature. Becoming a pianist or violist does depend on a great deal of teaching and practice, but this is the acquisition of a very specialized physical skill. Meanwhile, acquiring the musical conventions of your culture is no more demanding than mastering your native language (Bigand and Poulin-Charronnat, 2006).

When considering general musical ability (rather than formal musical training), it seems that musical and linguistic development continues on parallel tracks after the first year of life. Between 2–3 years of age, toddlers gain competence with the syntax of their native language (e.g., Höhle et al., 2001) and with the syntax of their culture’s music (in Western music, knowledge of key membership and harmony; Corrigall and Trainor, 2009). This is not complete syntactic competence, however: at age 5, semantics and syntax are still interdependent for children (Friederici, 1983; Brauer and Friederici, 2007), although by age 6, children appear to have mastered the basic syntax of their native language (Scott, 2004; Nuñez et al., 2011). Similarly, knowledge of musical key membership seems to have developed by about 5 years of age (Trehub et al., 1986; Trainor and Corrigall, 2010). However, while 5 year old children show adult-like electrophysiological responses to incorrect chords (Koelsch et al., 2003), they fail to detect a change in a melody that implies a different harmony (Trainor and Trehub, 1994). This requires a more nuanced understanding of harmony that does not fully develop until age 7 (Trainor and Trehub, 1994) when their knowledge of their native tonal structure is comparable to an adult’s (Speer and Meeks, 1985; McMullen and Saffran, 2004). Similarly, syntactic learning of more complex linguistic constructions continues through age 10 (Friederici, 1983). Children’s pitch discrimination abilities also reach adult levels between 8–10 years of age (Werner and Marean, 1996), and by age 12, their sensitivity to implied harmonies reaches adult levels (Costa-Giomi, 2003).

The similarities of these timelines are remarkable, especially given that all of these studies investigated children from Western cultures, which typically prioritize language learning in school curricula while placing less emphasis on music. Indeed, children given music lessons reach musical developmental milestones sooner than children without music lessons (for a review, see Trainor and Corrigall, 2010). Given that musical development keeps pace with linguistic development even in the face of limited musical instruction, it becomes even clearer that music acquisition is neither especially slow nor effortful.

The evidence discussed so far focuses on music perception, but what about the ability to *make* music? Although Western culture draws a separation between musicians and non-musicians, this has not always been the case and is certainly not true for many non-Western societies where singing (and dancing) are as integral to the community and to the culture as speaking. Because of the relative unimportance of music and singing in Western culture, however, little attention has been paid to the development of singing abilities. Nevertheless, the few studies that have been done suggest that the development of singing ability follows the same trajectory across cultures, with interesting parallels to aspects of language development. For instance, 2 year olds can repeat brief phrases that have an identifiable rhythmic and melodic contour (Dowling, 1999), which is about the same time that young children start to produce short linguistic utterances (Friederici, 2006; Gervain and Mehler, 2010). Three-year olds will mix elements of songs from their culture with their own idiosyncratic vocal improvizations, singing “outline” songs that follow the general contour of culture-specific melodies (Moog, 1976; Davidson, 1994; Hargreaves, 1996), which may be akin to the tendency for 2–3 year olds to eliminate function words, but not the content words in speech (Gerken et al., 1990). Singing ability continues to improve until about 11 years of age (e.g., Howard et al., 1994; Welch, 2002), though ability improves faster and to a greater extent in cultures that emphasize singing (Kreutzer, 2001; Welch, 2009).

Trehub and Trainor (1993) have reasoned that, if the evidence were to show that musical development exhibits “a developmental pace characteristic of innate guided learning, this would revive interest in its biological significance” (317). Music acquisition does, remarkably, keep pace with linguistic development, even in Western cultures where it is not on an equal educational footing with language. The parallels are even closer in cultures that emphasize music (cf. Kreutzer, 2001). If musical development appears to be slower and more effortful than language acquisition, it seems to be largely a product of culture, not biology.

In sum, infants’ learning of sound structure is based strongly on the musical aspects of sound. This is true in infant directed auditory input, where musical features are exaggerated in both speech and song, and in incidental statistical learning, which relies strongly on musical features like rhythm (especially early in development). These types of exposure are not independent – in fact, statistical learning can be enhanced in the context of infant directed speech (Thiessen et al., 2005) – but the development of both music and speech rely heavily on the musical aspects of children’s environments.

Why is music a part of every human culture? Because language is initially transmitted to children through speech, music cognition may play a strong adaptive function, enabling children’s linguistic skills to mature more rapidly. Arguments for innate language ability often appeal to the “poverty of stimulus” problem (Chomsky, 1980): language is too complex for children to learn based on positive evidence alone. Along with social cues such as facial expressions and physical gestures, the musical features of language may help surmount the “poverty of stimulus” and provide a richer context for language induction. From a developmental perspective, the progression is clear: first we play with sounds; then we play with meanings and syntax. It is our innate musical intelligence that makes us capable of mastering speech. Music as an art-form may develop from this initial entanglement: it may enable us to continue to explore and exploit features of music cognition that language does not prioritize.

## Musicians and Later Language Acquisition

If, as we propose, music cognition plays a strong role in early language acquisition, we would expect that musical training would correlate with improvements in language learning later in life. In fact, musical training and expertise confer many linguistically relevant advantages (for recent reviews, see Kraus and Chandrasekaran, 2010; Besson et al., 2011; Strait and Kraus, 2011; Slevc, 2012). These include advantages in “low-level” sound processing: musicians show a more faithful brainstem representation of pitch (measured using the frequency-following response or FFR) than non-musicians (Bidelman et al., 2011) presumably as a result of feedback pathways from the cortex to the brainstem (see Kraus and Chandrasekaran, 2010, for a review). This is not only true for musical stimuli, but also for spoken syllables in one’s native language (Musacchia et al., 2007) and in a foreign tone language (Wong et al., 2007). Importantly, these low-level enhancements have practical advantages; for example, musicians are better able to perceive speech-in-noise than non-musicians (Parbery-Clark et al., 2009; Bidelman and Krishnan, 2010). This enhancement extends even to older adults, where musical training seems to protect against the typical decline in the ability to perceive speech in noisy environments (Parbery-Clark et al., 2011).

Musical training also leads to advantages in the processing of prosody: musicians show greater sensitivity than non-musicians to emotional prosodic cues (Thompson et al., 2004; Lima and Castro, 2011) and better detection of subtle prosodic variations at the end of utterances in both their native and in a foreign language (Schön et al., 2004; Marques et al., 2007). Musical training is also associated with better discrimination of subtle timing contrasts in both native and foreign speech (Marie et al., 2011; Sadakata and Sekiyama, 2011). These advantages, too, have practical advantages, for example, in the ability to perceive and learn second language sound structures (Slevc and Miyake, 2006; Lee and Hung, 2008; Delogu et al., 2010).

Linguistic benefits of musical training are not confined to adult musicians: children taking music lessons also show linguistic enhancements relative to their non-musician peers. Like adults, they are better at detecting subtle prosodic variations at the end of utterances (Magne et al., 2006). They also show enhanced passive and active syllable processing, especially voice onset time, a critical ability in distinguishing consonants (Chobert et al., 2011), and show advantages in reading development and phonological awareness (e.g., Lamb and Gregory, 1993; Anvari et al., 2002; Forgeard et al., 2008). Music lessons in children can even enhance pre-linguistic communicative development (i.e., communicative gesturing; Gerry et al., 2012).

One might argue that these advantages reflect innate differences instead of being an effect of musical training itself. While there is relatively little longitudinal data thus far, there is evidence that differences in brain anatomy associated with musicians (e.g., the size of the corpus callosum) can already be seen in the brains of children who have taken 30 months of music lessons, despite showing no differences prior to starting music lessons (Schlaug et al., 2009a). Similarly, longitudinal studies of children assigned to music or painting lessons show that musical training benefits reading abilities (after only 6 months of lessons) and speech segmentation (after 2 years of lessons), as evidenced by both behavioral and electrophysiological measures (Moreno et al., 2009; François et al., in press).

Patel’s (2011) OPERA hypothesis proposes that these benefits of musical training result from overlapping language/music networks, the fact that music involves precise auditory processing, emotional engagement, repetition (i.e., practice), and high attentional demands. Evidence that attending to the musical features of speech is an effective language learning strategy may have implications for adult learning and recovery as well. For example, a focus on musical aspects of speech may improve second language acquisition (cf. Slevc and Miyake, 2006) and musically based therapy may effectively treat developmental and acquired language deficits (see, e.g., Schlaug et al., 2009b).

Although these findings cannot establish a direct link between music and language learning in infants, they are an anticipated outcome of our hypothesis. In addition, the extensive volume of work enhances the view that music and language share many similar properties – something we might expect infants to observe, especially before they are attuned to speech’s referential meaning.

## Challenges and Caveats

It is, of course, somewhat controversial to claim that speech is processed as a special form of music. Many have claimed that speech and music are separable modular systems (e.g., Peretz and Coltheart, 2003). Such a separation has even been claimed to be innate, given evidence that infants show left hemispheric lateralization for speech perception and right hemispheric lateralization for frequency perception (Dehaene-Lambertz et al., 2002, 2010). However, these hemispheric asymmetries may reflect cortical specialization for more general auditory properties rather than specificity for speech or music *per se* (e.g., a left hemisphere specialization for rapid temporal processing; Zatorre et al., 2002; Hickok and Poeppel, 2007). Although the hemispheric division of labor is likely not straightforward (Telkemeyer et al., 2009; McGettigan and Scott, 2012), the insight remains that hemispheric differences likely reflect processing asymmetries in aspects of auditory processing rather than specializations for speech or music.

Furthermore, other work does not support this early specialization for language, showing either no lateralization for speech stimuli (e.g., Dehaene-Lambertz, 2000; Kotilahti et al., 2010), or even *right* hemispheric lateralization for speech (Perani et al., 2011) that closely parallels activation to music in an earlier study (Perani et al., 2010). These findings suggest that hemispheric specialization emerges over the course of development. In further support for this idea, early damage to the right hemisphere tends to lead to more severe later language problems than early damage to the left hemisphere (Bates et al., 1997).

A second powerful argument for the neural separability of music and language comes from the dissociation of musical and linguistic abilities sometimes seen in brain damaged patients. Musical deficits can occur without linguistic deficits; in particular, amusics have great difficulty with pitch processing yet typically seem to have normal language abilities (e.g., Ayotte et al., 2002; Peretz, 2006). However, recent work suggests that amusics do in fact have difficulty with aspects of prosodic perception (Liu et al., 2010) and with aspects of phonological and phonemic awareness (Jones et al., 2009). It may seem surprising that these impairments go unnoticed among amusics, however this likely results from the multiple cues to meaning in spoken language, which allow for successful processing of conversational speech even with impaired pitch processing.

Linguistic deficits can accompany preserved musical processing as well; for example, the Russian composer Vissarion Shebalin continued to compose after a series of strokes left him with profound language deficits (Luria et al., 1965; see also Basso and Capitani, 1985; Tzortzis et al., 2000). Such cases provide strong evidence for some degree of music/language separability, however, they may reflect damage to abilities that have become specialized and neurally separated over development (cf. Karmiloff-Smith, 1995). Supporting this claim, all reported cases of preserved musical processing accompanying linguistic deficits involve professional musicians, who one might expect to show a relatively higher degree of specialization (Tzortzis et al., 2000). These cases also reflect a variety of deficits; cases of preserved musical *sound processing* in the presence of linguistic *sound processing* deficits are elusive at best (e.g., Mendez, 2001; Slevc et al., 2011). While there is little data on language deficits without musical deficits in non-musicians, some evidence does suggest that aphasia in non-musicians may also be accompanied by deficits in aspects of pitch and harmonic processing (Frances et al., 1973; Tallal and Piercy, 1973; Patel et al., 2008).

One might also object to the thesis that language acquisition is inherently musical based on a wide range of evidence that language acquisition is inherently a *social* process. For example, the effectiveness of the sorts of infant directed communication discussed above may be in part musical, but clearly one of the main reasons for the capture of attention is that infant directed communication is highly socially and emotionally expressive (Trehub, 2003). Music is well suited for this sort of communication, however, it is not *only* music that can have these effects; sign language speakers also use infant directed *sign language*, and both deaf and hearing infants prefer this “sign motherese” to adult directed sign language (Masataka, 1996).

The fact that deaf children are able to learn sign languages (at least when receiving appropriate input) may seem especially problematic for the view that musical perception underlies language learning. However it may be that the very aspects of music that are advocated here – especially its nature as a flexible and constantly evolving form of expression – make it well suited to adapt even to different modalities. In particular, the rhythmic and expressive nature of gesture and sign babbling (e.g., Petitto and Marentette, 1991; McClave, 1994) might be a sort of visual parallel to the music of speech.

There are many other questions that need to be addressed before the nature of music and language and its entanglement in the brain (infant or adult) can be satisfactorily resolved. As noted earlier, there are almost no studies of infant timbre processing, nor has much work investigated timbre processing in dyslexia, SLI, or other language disorders. Testing timbre discrimination, especially of instrumental attacks using both native and non-native instrumental timbres, would be informative: if it were shown that phonemic processing was innately separate from the processing of musical timbre, it would raise substantial questions about our claims. Likewise, there is only a small (but growing) body of work on the existence of subtle linguistic deficits in amusia and of subtle (or not so subtle) musical deficits in aphasia. Research on musical development between 12–24 months of age is scarce, perhaps simply because infants of that age are difficult to test; closing this gap would contribute to our understanding of the co-development of music and language. Finally, more research into music and linguistic processing in non-Western cultures is needed. Until research addresses language and music from a broad cross-cultural context, any claims must be circumscribed within a specific cultural context.

## Conclusion

A child’s first words are eagerly awaited not only as a cognitive milestone, but as a bond with the adult world – one that heralds the full measure of human thought and expression. But that is not how language cognition begins. For the first year of life, babies hear language as an intentional and often repetitive vocal performance. They listen to it not only for its emotional content but also for its rhythmic and phonemic patterns and consistencies. As Newham says: “…whereas the verbal infant will later organize such sounds according to the rules of the dictionary, the baby, not yet familiar with such a scheme, arranges them according to an intuitive, creative, and innate sense of pitch, melody, and rhythm in a fashion directly akin to the composition of music.” (Newham, 1995–1996, p. 67). As sounds are mapped onto meaning, language’s referential function increasingly commands the child’s attention because of its social importance. However, during the first year of life, a different type of listening prevails. Music as an art-form may be a way of prolonging this earlier period, when we encountered the world as a concert and sentences were merely sounds^4^.

So while music and language may be cognitively and neurally distinct in adults, we suggest that language is simply a subset of music from a child’s view. By this account, music, and language are examples of emergent modularity (see, e.g., Karmiloff-Smith, 1995; Johnson, 2010; Johnson, 2011) arising from a common cognitive root.

Is it appropriate to call this common root “musical”? Throughout the world, normally hearing infants are taught language through speech. Both music and speech involve “creative play with sound” and require an attention to acoustic features. The primary difference between them is that the speech is referential. However, as the cited studies have demonstrated, that is a distinction that infants are not yet capable of making in the first year of life.

Would it be better to call the infant’s listening skills “proto-musical”? If a mother sings a lullaby to her child on the first day of life, no one would expect the child to understand the lyrics; but we might reasonably deduce that the child recognizes the mother’s repetition and soothing prosody – and falls asleep accordingly. If we define music as “creative play with sound,” then the evidence suggests that musical engagement is a great way of describing of what infants are doing. As long as the definition of music is the elemental one we are advocating, this terminology does not impose adult categories on the young. As we develop, our musical intelligence is refined based on cultural norms and individual taste and our cognition of music and language become more modular. However, we never lose our innate capacity to treat *any* sound imaginatively. Other than culturally specific features, it is hard to understand what would separate “proto-music” from “music;” any dividing line would likely not translate across cultures and risks sowing confusion.

Music is often described as a universal language but it is neither: musical universals across eras and cultures have been stubbornly difficult to find; you cannot order a soda or use the future tense without vocabulary and syntax. However, it may *feel* like a universal language because, for normally developing humans, it underlies the way that we acquire language: as “creative play with sound,” it directs our attention to and amplifies the features of speech that we were paying attention to *before* we were listening for referential meaning. Human creativity, aural abilities, and a desire to communicate underlie both music and language. Listening to music may give us insights into how language sounds to us before we understand it – and how we experience our world before we have words.

## Authors’ Note

The authors wish thank the following for their helpful discussions and advice: Rich Baraniuk, Jonathan Berger, C. Sidney Burrus, J. Todd Frazier, Robert Gjerdingen, Erin Hannon, David Huron, Nina Kraus, Stephen McAdams, Anirrudh Patel, Elizabeth Redcay, Jason Reitman, Kay Kaufman Shelemay, JP Slavinsky, Trent Walker, and Lawrence Zbikowski. We would also like to wish to express our gratitude to the following at Rice University for their support of our project: Caroline Levander, Vice Provost for Interdisciplinary Initiatives, Jan Odegard, Executive Director of the Ken Kennedy Institute, and Robert Yekovich, Dean of the Shepherd School of Music.

## Conflict of Interest Statement

The authors declare that the research was conducted in the absence of any commercial or financial relationships that could be construed as a potential conflict of interest.

## Footnotes

^1^A sound’s envelope consists of an attack, sustain, and decay, measured as changes in amplitude over time. The envelope creates the basic unit of musical rhythm: the sound’s starting point and its duration. Frequency denotes the oscillations of a sound wave, measured in cycles per second. A sound’s spectrum describes the shape of a sound wave. The sine wave is the basic shape. Deformations create “overtones.” A “harmonic” spectrum involves a periodic fundamental frequency and overtones or partials in simple arithmetic ratios to the fundamental. In human hearing, harmonic sounds produce a stable sense of pitch. Enharmonic sounds include *any* sound that is *not* periodic and in which the overtones are not in simple arithmetic ratios. Depending on the complexity and lay-out of the partials, enharmonic sounds can produce varying degrees of pitch perception, from the “blurry” to the “noisy.”

^2^The Oxford English Dictionary defines language as: “the method of human communication, either spoken or written, consisting of the use of words in a structured and conventional way” (Language, 2012a). Merriam-Webster defines it as “the words, their pronunciation, and the methods of combining them used and understood by a community” (Language, 2012b).

^3^Though note that speech sounds that do not exist in the native language, such as Zulu click contrasts for English speakers, remain easily distinguishable by adults (Best et al., 1988).

^4^Note the similarity to claims that the evolution of music and language are closely linked (Brown, 1999; Mithen, 2006; Panksepp, 2009): these authors suggest that language may have developed from more primitive vocalizations – we “sang” before we spoke. It thus may be the case that, with respect to the development of music and language, ontogeny recapitulates phylogeny (cf. Gould, 1977).
